# Branched Polyacrylonitrile Enabling Highly Lithium-Ion-Conductive Polymer Plastic Crystal Electrolytes

**DOI:** 10.1021/acsmacrolett.5c00576

**Published:** 2025-10-08

**Authors:** Xin Liu, Junlong Yang, Feichen Cui, Zixiao Wang, Honglu Huang, Yipeng Zhang, Hua Liu, Chao Xu, Jiajun Yan

**Affiliations:** School of Physical Science and Technology, 387433 ShanghaiTech University, Shanghai 201210, China

## Abstract

Advancing the development of high-performance solid-state electrolytes is critical for realizing next-generation lithium metal batteries. Among promising candidates, polymer–succinonitrile composites have emerged as effective polymer plastic crystal electrolytes, demonstrating enhanced electrochemical performance. However, further improvements are needed to meet practical application requirements. In this study, we report a novel strategy for synthesizing electrochemically stable branched polyacrylonitrile through controlled/living branching radical polymerization, employing 2-chloroacrylonitrile as an innovative inibramer. The unique branched architecture of the resulting polymer facilitates continuous pathways, enabling rapid lithium-ion transport when incorporated in polymer plastic crystal electrolytes. Electrochemical characterization reveals substantial improvements in both ionic conductivity and stability compared to conventional linear counterparts. These findings highlight the pivotal role of polymer architectural design in optimizing ion transport within solid electrolytes, offering new opportunities for developing safer and more efficient energy storage devices.

## Graphical abstract

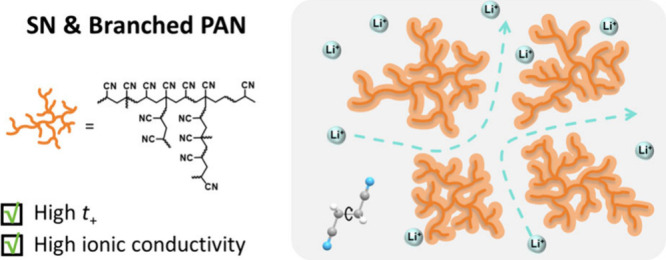

Lithium batteries (LBs) dominate modern energy storage in numerous applications due to their superior power density and lightweight characteristics, powering portable electronic devices and electric vehicles.^1–3^ However, conventional liquid electrolytes are plagued by critical safety issues such as leakage, flammability, and lithium dendrite growth under the backdrop of increasingly stringent safety requirements,^4,5^ driving the urgent need for safer and more efficient alternatives.

Solid-state electrolytes have emerged as a promising solution in electrolyte technology development, with enhanced safety performance, simplified production processes, and improved sustainability.^6–8^ Among them, polymer plastic crystal electrolytes (PPCEs) have garnered widespread attention due to their high ionic conductivity comparable to liquid electrolytes, excellent electrode compatibility, and robust mechanical properties.^9–12^ Succinonitrile (SN) is considered an ideal plastic crystal material for lithium-ion battery electrolytes, owing to its outstanding dissolution of various lithium salts in the plastic crystal phase while maintaining high ionic conductivity.^13^ The introduction of lithium bis­(trifluoromethanesulfonyl)­imide (LiTFSI) into the SN system enables an ionic conductivity of up to 10^–4^ S cm^–1^ at room temperature, showcasing its tremendous potential in the field of plastic crystal electrolytes for lithium-ion batteries.^14^ This material successfully bridges the gap between liquid and solid electrolytes, combining superior ion transport with enhanced safety and stability, paving a new path for lithium-ion battery technology development.^15–17^

Polyacrylonitrile (PAN) contains polar and electron-withdrawing nitrile groups, conferring excellent mechanical strength, thermal stability, electrochemical stability, and flame retardancy, making it an ideal material for solid-state electrolytes.^18,19^ The nitrile groups in PAN also coordinate with Li^+^, promoting lithium salt dissolution and inhibiting lithium dendrite growth while conducting lithium ions.^20,21^ Moreover, the molecular structure of polyacrylonitrile creates channels for ion transport, facilitating rapid ion movement and enhancing ionic conductivity.^22^ Helms and co-workers^23^ discovered that PAN-doped SN electrolytes formed a plastic crystal-polymer high-entropy interface, where selective ion distribution and enhanced Li^+^ diffusion arose from PAN-induced increases in molar volume and bond rotation frequency. Further study by Bottke and co-workers^24^ confirmed that SN–LiTFSI–PAN composites outperform SN–LiTFSI mixtures with thermoplastic polymers such as poly­(ethyl cyanoacrylate), poly­(ethylene oxide) (PEO), and poly­(*N*-vinylpyrrolidone) by exhibiting higher Li-ion conductivity and lower activation energies, attributed to synergistic nitrile group interaction that creates a homogeneous ion-conduction environment.

Branched polymers, a type of macromolecule with a unique three-dimensional spherical structure, exhibit characteristics distinctly different from traditional linear molecules, including low viscosity, excellent solubility, and tunable functionality.^25–28^ Their branching nodes disrupt polymer chain ordering, reducing crystallinity and enhancing ionic conductivity in electrolytes.^29–32^ For instance, branched PEO exhibits lower chain entanglement than linear PEO, yielding larger free volume and amorphous regions.^33^ In the SN and LiTFSI composites, these amorphous domains foster continuous high-entropy interfaces, enabling superior Li^+^ conduction. Meanwhile, the increased freedom of segment movement brought about by the free volume improves ion hopping.^34^ In contrast, the random coil morphology of linear polymers limits the connectivity of amorphous interfacial regions, impeding ion transport (Scheme 1a).^35,36^

**Scheme 1:**
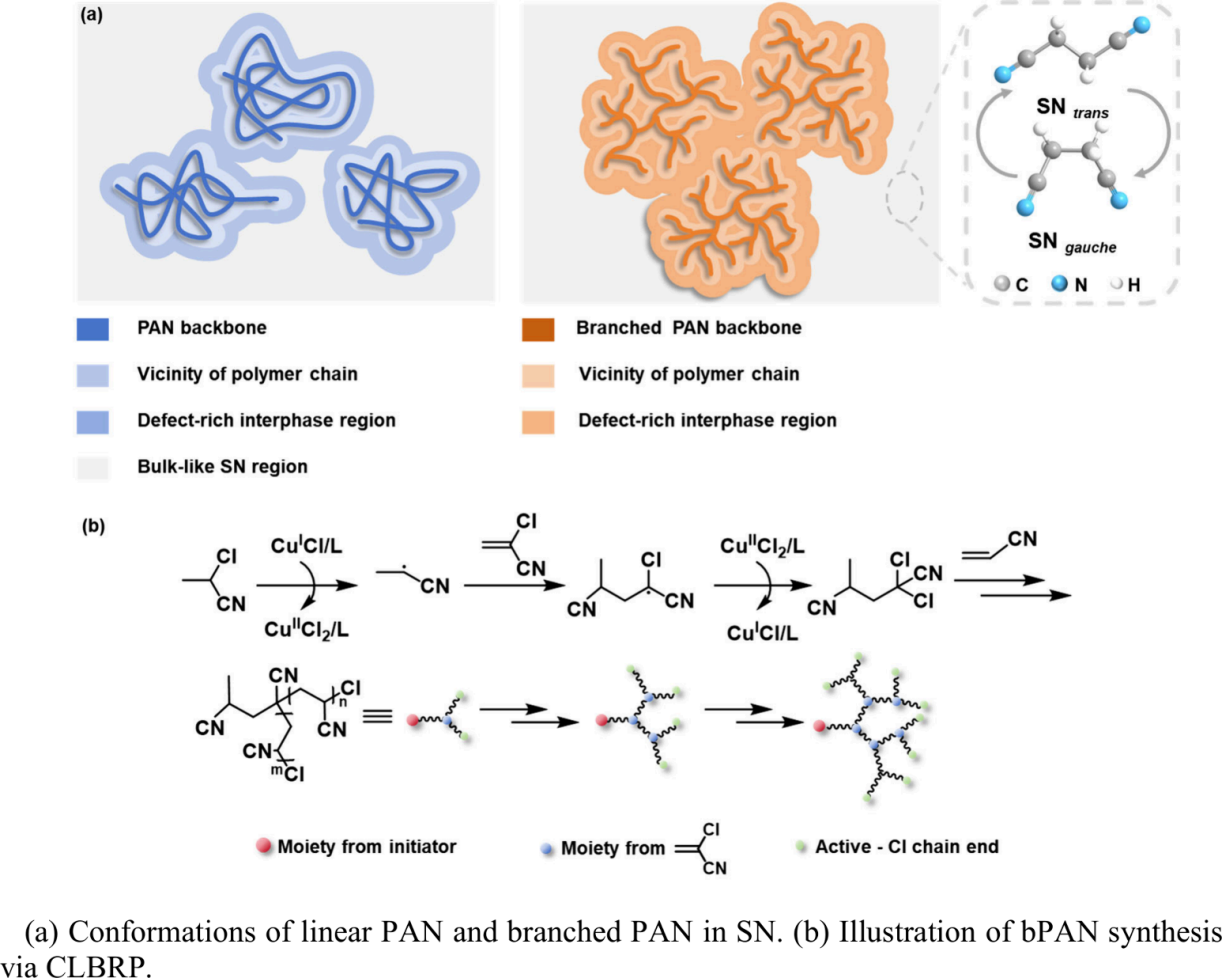
Polymer Plastic Crystal Electrolyte with Branched Polyacrylonitrile (bPAN)

In conventional approaches, branched polymers are typically synthesized via grafting strategies, where linear polymer chains grow in situ under the action of macroinitiators. However, this methodology inherently presents two critical limitations. First, the absence of precise growth regulation and site-specific initiation mechanisms hinders hierarchical architecture construction of branched polymers through macroinitiators, simultaneously compromising control over molecular weight distributions and resulting in products with high dispersity.^37^ Second, conventional polymerization protocols inevitably require either inimers or backbone polymers containing chemically labile ester linkages, a structural vulnerability that significantly constrains material stability and potential applications.^33^ Zhong and co-workers^38,39^ recently proposed controlled/living branching radical polymerization (CLBRP), which integrates branching motifs into macroinitiators during chain extension to create hierarchical branched structures.

Building upon Zhong’s CLBRP,^39^ we herein developed a synthetic strategy using 2-chloroacrylonitrile (CAN) as an innovative inibramer in CLBRP to synthesize branched polyacrylonitrile to address the challenges of conventional synthesis of branched polymer. As illustrated in Scheme 1b, our approach initiate polymerization from 2-chloropropionitrile (CPN),^40,41^ with CAN serving dual critical functions: (1) CAN’s *sp* ^2^ C–Cl bond exhibits higher dissociation energy than traditional initiators, preventing premature activation by conventional ATRP catalysts;^41^ (2) while maintaining copolymerizability with AN, it forms two *sp* ^3^ C–Cl bonds upon reacting with propagating radicals, creating reactivatable branching points analogous to 2,2-dichloronitrile species.

In our initial attempt, a one-pot copolymerization of AN and CAN under ATRP conditions using a monomer feeding ratio of AN/CAN = 90/10 exhibited negligible conversion (Table 1, Entry 1). While increasing the AN/CAN ratio to 500/8 according to the literature^38^ enabled the polymerization, AN conversion remained low (Entry 2). Subsequently, we adopted a semibatch strategy, i.e., the slow addition of CAN to the polymerization solution at controlled feeding rates, to modulate cross-propagation reactions (Entries 3–10).^39^ Testing began with a 95/5 feeding ratio (Entry 3), followed by systematic evaluation of the literature-reported 500/8, 300/8, and 200/8 ratios to optimize branching density control.

**Table 1:** CLBRP Synthesis of bPAN^a^

Entry	[AN]_0_/[CAN]_0_ /[CPN]_0_	Feeding rate (eq/h)	Reaction time (h)	Conv._AN_(%)	*M*_w_ ^c^(kDa)	*M*_n_ ^c^ (kDa)	*Đ* ^c^	*S*_n_ ^d^
1	90/10/1	-^b^	40	-	-		-	-
2	500/8/1	-^b^	40	1.53	322	153	2.11	9.39
3	95/5/1	0.8	40	4.15	35.3	23.2	1.52	5.77
4	200/8/1	0.2	45	12.8	31.2	19.9	1.56	12.4
5	200/8/1	0.4	40	7.78	35.1	19.4	1.81	9.63
6	300/8/1	0.2	45	14.2	30.8	23.4	1.32	18.4
7	300/8/1	0.4	40	8.81	60.2	25.4	1.83	17.6
8	500/8/1	0.2	45	20.1	31.5	23.4	1.35	31.5
9	500/8/1	0.4	40	18.7	23.4	20.3	1.59	28.6
10	500/8/1	0.8	40	5.32	70.5	41.4	1.70	21.4

a Conditions: [CPN]_0_/[CuCl_2_]_0_/[Me_6_TREN]_0_ = 1/0.01/0.03 at room temperature. [AN]_0_ = 15.19 M in dimethyl sulfoxide. Me_6_TREN: tris­[2-(*N*,*N*-dimethylamino)­ethyl]­amine; Conv.: conversion; *M*_w_: weight-average molecular weight; *M*_n_: number-average molecular weight; *Đ*: dispersity; *S*_n_: spacer value; NMR: nuclear magnetic resonance.

b One-pot copolymerization.

c Measured by a size exclusion chromatography–multiangle light scattering system eluted with *N*,*N*-dimethylformide under a 100% recovery assumption for known injection concentrations.

d Measured by ^1^H NMR using eq S3.

The degree of branching was evaluated using the spacer value (*S*_n_), defined as the average number of monomer units inserted between two adjacent branch points.^42^ We first evaluated the *S*_n_ measurement by ^1^H NMR and quantitative ^13^C NMR on one of the bPAN synthesized via CLBRP (Entry 8, Figures S8 and S10). The two techniques gave *S*_n_ of 31.5 and 30.9 (eqs S2 and S4), respectively, demonstrating consistency. We thereby proceeded with the more efficient ^1^H NMR measurement for the other samples (Table 1, Table S1). Overall, higher degrees of branching were achieved from lower [AN]_0_/[CAN]_0_ feeding (Entries 4 and 5). However, the AN conversion was also limited in these cases.

We further evaluated the influence of feeding rate. As the feeding rate slightly improved the degree of branching, a higher feeding rate suppressed the incorporation of AN (Entries 4–10), as predictable from the one-pot experiments (Entry 2). Therefore, a higher or faster feeding of CAN inhibited the incorporating of AN while promoting branching, which is attributable to their distinct reactivity ratios (*r*_AN_ = 0.31 and *r*_CAN_ = 3.25).^43^

The optimal condition for bPAN synthesis with an AN/CAN feeding ratio of 500/8 at 0.2 eq/h gave a high acrylonitrile conversion of 20.1% while preserving the targeted branched architecture with *S*_n_ = 31.5, concurrently yielding a low *Đ* of 1.35. Figure S1 shows that the propagation of AN occurs at a nearly constant rate. The bPAN produced in this protocol was then used for further PPCE studies.

To evaluate the effectiveness of bPAN in improving ionic conductivity, we prepared solid-ion conductors (SICs, Figure S12) and assembled coin cells as illustrated in Figure S13. The structural similarity allowed PAN and bPAN to readily dissolve in molten SN. We then added LiTFSI. The SICs without polymer, with linear PAN, and with bPAN are annotated as SN, SN-PAN, and SN-bPAN, respectively.

We first investigated the thermomechanical properties of the three types of electrolytes in rheometric measurements (Figure 1a). Below 55 °C, *G*′ of all samples consistently exceeded *G*″, indicating viscoelastic solid-like behaviors. The moduli followed the order SN < SN-bPAN < SN-PAN, suggesting that the polymer incorporation served as an elastic component to enhance the mechanical properties. Additionally, the bPAN-SN electrolyte exhibits a lower room-temperature modulus than its linear counterpart, attributable to the reduced physical entanglement of the branched macromolecules in SN.^44,45^

**Figure 1:**
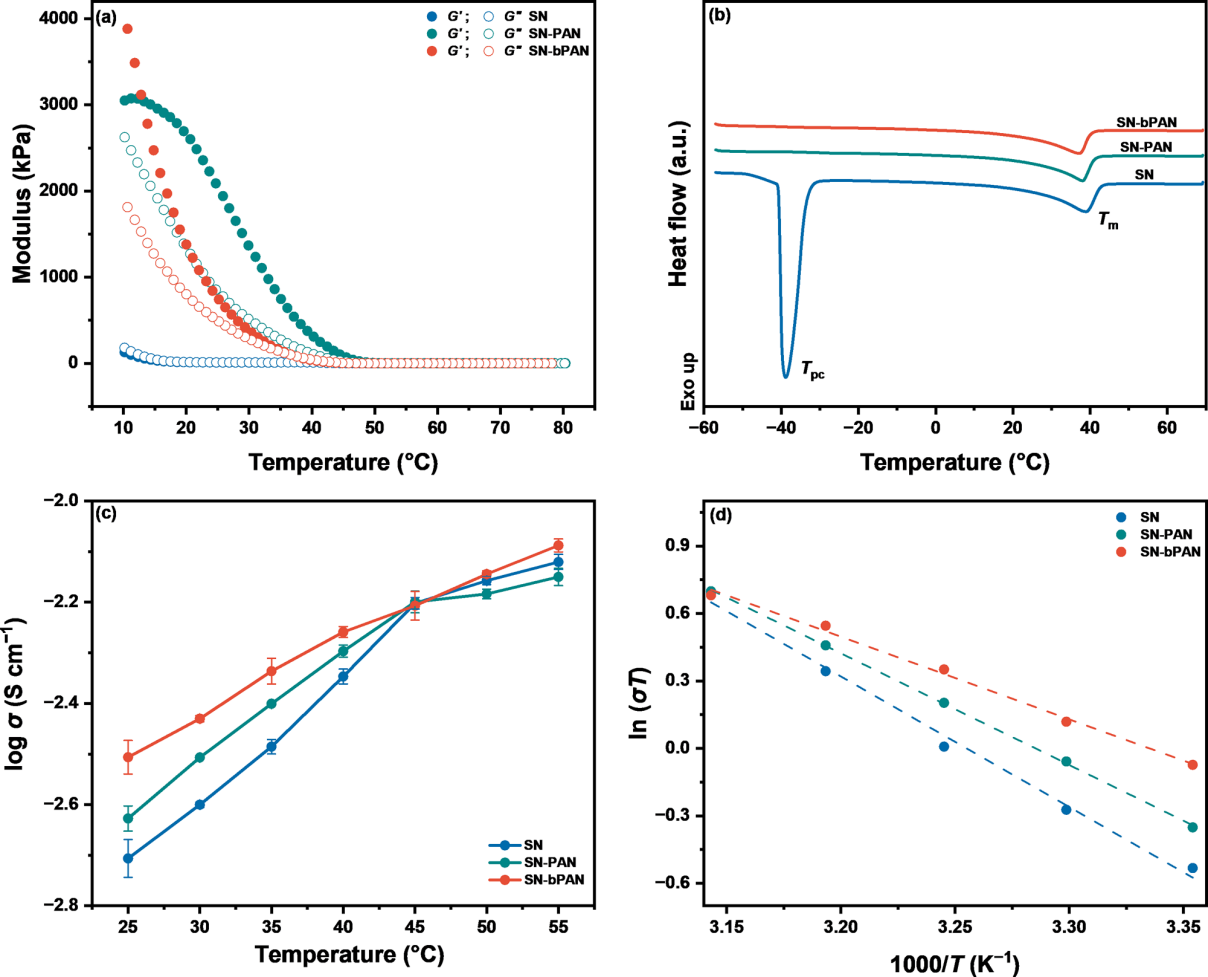
Characterization of SICs. (a) Storage (*G*′) and loss (*G*″) moduli as a function of temperature; (b) DSC curves; (c) temperature-dependent ionic conductivities (error bars: standard deviation, *n* = 3); and (d) Arrhenius plot. SN: 0.50 M LiTFSI-doped SN; SN-PAN: 0.50 M LiTFSI-doped SN/7.5 wt % PAN composite; and SN-bPAN: 0.50 M LiTFSI-doped SN/7.5 wt % bPAN composite.

At the solid–solid phase transition temperature (*T*_pc_), SN undergoes a transition from a fully ordered, all-*gauche* crystalline state to a plastic crystalline state where *trans*-isomers are present as rotational motions start. The *trans*-isomer presented as an impurity and leads to an increased degree of disorder, resulting in faster molecular diffusivity within materials facilitating Li^+^ transfer.^46–48^ We used differential scanning calorimetry (DSC) to determine the potential change of the *T*_pc_ and the melting temperature (*T*_m_) with the addition of PAN (Figure 1b).^47,49^ The SN exhibited a *T*_pc_ of −38 °C, which was marginally higher than that of pure SN (*T*_pc_ = −40 °C). In contrast, both SN-PAN and SN-bPAN were devoid of any *T*_pc_. The rise of *T*_pc_ in the SN SIC was a result of the interaction between the Li salt and SN. Meanwhile, the absence of *T*_pc_ in the polymer-doped samples indicated a loss of order even at very low temperatures.^50^ The *T*_m_ of all three samples are similar, with SN-PAN (*T*_m_ = 38.6 °C) and SN-bPAN (*T*_m_ = 37.7 °C) slightly lower than that of SN (*T*_m_ = 39.6 °C). This drop in *T*_m_ further suggested an increase in the amorphous portion at room temperature, which in turn enhances ionic conductivity.^51^ The observed decrease in *T*_m_ can be attributed to the thermodynamic alterations induced by component mixing, particularly considering the presence of nitrile groups in both SN and PAN. The mixture of SN and PAN has a significantly reduced plastic–crystalline transition enthalpy as reported previously,^24^ suggesting effective miscibility between these components.^45,52–54^

We investigated the ionic conductivity of the SICs using electrochemical impedance spectroscopy (EIS) in coin cells (Figure 1c). Throughout the testing temperature range, SN-bPAN exhibited the highest ionic conductivity. At 25 °C, the ionic conductivities of SN, SN-PAN, and SN-bPAN were measured to be 1.97 × 10^–3^, 2.36 × 10^–3^, and 3.12 × 10^–3^ S·cm^–1^, respectively. The ionic conductivity of the SN-bPAN composite exhibits a notable improvement at temperatures approaching ambient conditions. When SN molecules undergo *trans*–*gauche* interconversion around the central C–C bond, disruptions in the local solid solvation environment cause Li^+^ to hop from their initial solvation sites to neighboring ones.^48,52–54^ Previous reports showed SN had larger molar volume at the PAN-SN plastic crystal-polymer high entropy interphase, providing greater freedom for more frequent conformational interconversions, which facilitate the ion transport process and increase the diffusion coefficient of Li^+^.^55^ SN molecules far from PAN are predominantly in the *gauche* conformation, with few interconverting to the *trans* conformation. In our systems, compared to linear PAN, bPAN exhibits a lower degree of chain entanglement, resulting in more connected amorphous SN regions. This creates more continuous high-entropy interfaces within the composite, leading to a further increase in the ionic conductivity.^17^

The activation energy (*E*_A_) was extracted from the conductivity measurements (Figure 1d, Table S2), revealing values of 0.46, 0.40, and 0.29 eV for SN, SN-PAN, and SN-bPAN, respectively. The incorporation of PAN was observed to significantly reduce *E*_A_. This phenomenon can be attributed to the interaction of nitrile groups between PAN and SN, which effectively enhances structural disorder within the material, thereby lowering the energy barrier. Furthermore, the integration of bPAN yielded a more pronounced decrease in *E*_A_. This empirical evidence substantiates the hypothesis that branched architectures engender a continuous, high-entropy interphase, thereby optimizing ionic diffusion pathways and further diminishing *E*_A_.^56,57^

In the development of electrolytes for solid-state batteries, factors such as electrochemical stability and Li^+^ transference number (*t*_+_) are crucial. Linear sweep voltammetry (LSV) revealed that SN-bPAN exhibits oxidation stability up to 5.18 V (vs Li^+^/Li) without detectable oxidation leakage current, attributed to the high electrochemical stability of SN-bPAN (Figure 2a). This electrolyte meets the application requirements for high-voltage cathodes.

**Figure 2:**
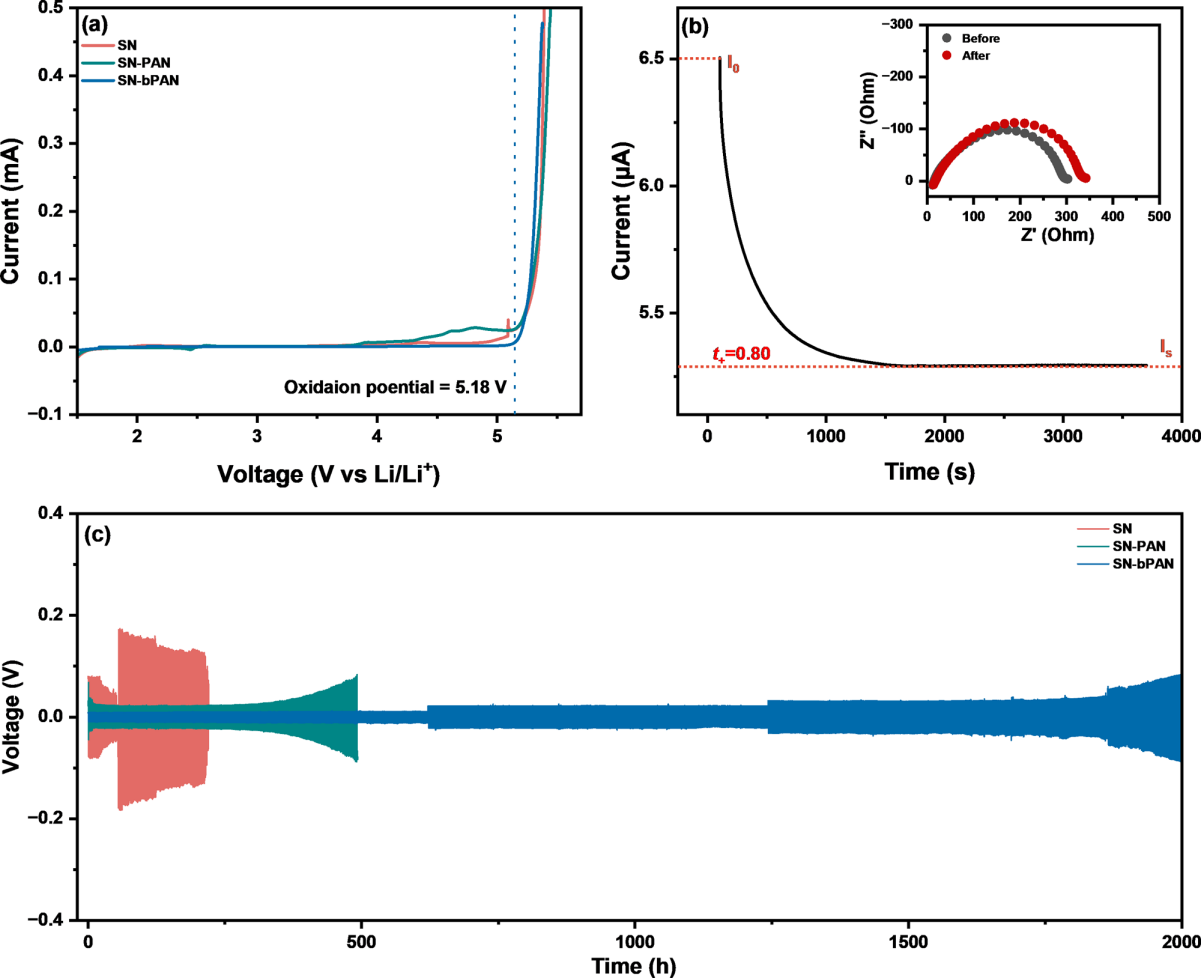
Electrochemical properties. (a) Linear sweep voltammetry of three SICs. (b) Li^+^ transference number of SN-bPAN. (c) Cycling performance of the symmetric Li–Li cells. SN: 0.50 M LiTFSI-doped SN; SN-PAN: 0.50 M LiTFSI-doped SN/7.5 wt % PAN composite; and SN-bPAN: 0.50 M LiTFSI-doped SN/7.5 wt % bPAN composite.

A higher *t*_+_ can effectively reduce electrode polarization and prevent harmful side reactions at the electrode. The *t*_+_ value for SN-bPAN was determined by integrating results from chronometry and electrochemical impedance analysis, resulting in a high *t*_+_ of 0.80, which is higher than that of SN and SN-PAN (Figure 2b, Figure S14). This value is attributed to the favorable dissociation of LiTFSI in the SN and bPAN composite. Additionally, PAN reduces the crystallinity of the material, thereby decreasing internal lattice obstructions to Li^+^ movement.^57^

Figure 2c illustrates the reversibility of lithium plating and stripping performance for the three SICs in a symmetric lithium battery at current densities of 50, 100, 150, and 200 μA·cm^–2^, with the current density increased every 600 h. In the symmetric cell using the SN electrolyte, the polarization potential remained stable for the first 18 h of cycling but then soared. The SN-PAN cell was stable for 300 h. This is plausibly due to spontaneous chemical reactions-nitrile polymerization catalyzed by lithium metal which severely damaged the electrolyte/electrode interface.^58^ In contrast, the symmetric cell with SN-bPAN exhibited a stable and low voltage profile over 1800 h, indicating a stable interface between the lithium metal and the SN-bPAN electrolyte. Considering its high ionic conductivity, wide electrochemical window, and excellent stability with lithium metal, the SN-bPAN electrolyte demonstrated satisfactory performance in lithium metal batteries.

In summary, we have developed an efficient synthetic strategy for electrochemically stable branched polyacrylonitrile via controlled/living branching radical polymerization, utilizing 2-chloroacrylonitrile as a novel inibramer. The resulting branched polymer exhibits enhanced capability for promoting lithium-ion transport when incorporated into polymer plastic crystal electrolytes in comparison to its linear analog. This enhancement stems from the formation of continuous, three-dimensional ion conduction pathways enabled by the precisely engineered branched architecture. Our findings highlight the significant potential of polymer architecture engineering in the design of high-performance solid-state ion conductors, paving the way to overcome current limitations in lithium metal batteries, potentially enabling next-generation energy storage systems with enhanced ionic conductivity, improved electrochemical stability, and superior mechanical robustness.

## Supplementary Material



## Notes

Detailed experimental procedures, additional calculations, and characterization data (PDF)
