# Supplementary material for: Branched Polyacrylonitrile Enabling Highly Lithium-Ion-Conductive Polymer Plastic Crystal Electrolytes
Source: ACS Macro Lett. 2025 Oct 8;14(10):1594–601. doi: 10.1021/acsmacrolett.5c00576 (PMC12548345; doi:10.1021/acsmacrolett.5c00576)
Supplement: Supplementary file 1 [file mz5c00576_si_001.pdf]

Supporting Information

Branched Polyacrylonitrile Enabling Highly Lithium-Ion-Conductive Polymer Plastic Crystal Electrolytes

Xin Liu, Junlong Yang, Feichen Cui, Zixiao Wang, Honglu Huang, Yipeng Zhang, Hua Liu, Chao Xu, Jiajun Yan**

School of Physical Science and Technology, ShanghaiTech University, Shanghai 201210, China

Table of contents

Experimental Section 4

Materials 4

Synthesis of branched polyacrylonitrile (bPAN). (Table 1, Entry 8) 4

Characterization 5

NMR 5

SEC 6

Differential scanning calorimetry (DSC) 7

Rheological tests 7

Assembly of stainless steel–stainless steel (SS-SS) symmetric cells and electrochemical impedance spectroscopy (EIS) determination 7

Assembly of SS–Li asymmetric cells and linear sweep voltammetry (LSV) 8

Assembly of Li–Li symmetric cells, Li^+ transference number (t_+), and lithium stripping/plating 8

Table S1. Calculation of degree of branching. 10

Table S2. Calculation of EA. 10

Figure S1. AN conversion versus polymerization time in the copolymerization of CAN and AN (Table 1, Entry 8). 11

Figure S2. ^{1}H-NMR spectrum of bPAN (**Table 1**, Entry 2). 12

Figure S3. ^{1}H-NMR spectrum of bPAN (**Table 1**, Entry 3). 13

Figure S4. ^{1}H-NMR spectrum of bPAN (**Table 1**, Entry 4). 14

Figure S5. ^{1}H-NMR spectrum of bPAN (**Table 1,** Entry 5). 15

Figure S6. ^{1}H-NMR spectrum of bPAN (**Table 1**, Entry 6). 16

Figure S7. ^{1}H-NMR spectrum of bPAN (**Table 1**, Entry 7). 17

Figure S8. ^{1}H-NMR spectrum of bPAN (**Table 1**, Entry 8). 18

Figure S9. ^{1}H-NMR spectrum of bPAN (**Table 1**, Entry 9). 19

Figure S10. ^{1}H-NMR spectrum of bPAN (**Table 1**, Entry 10). 20

Figure S11. Quantitative ^{13}C-NMR spectrum of bPAN (**Table 1**, Entry 8). 21

Figure S12. Digital photographs of as-prepared 0.5 M LiTFSI SICs at room temperature: (a) SN, (b) SN-PAN, and (c) SN-bPAN. 22

Figure S13. Preparation of electrolytes and assembly of batteries. 22

Figure S14. Li^+ transference number (a) SN, (b) SN-PAN 23

Figure S15. The SEC traces for liner PAN. 23

Reference 24

Experimental Section

Materials

Dimethyl sulfoxide (DMSO, Greagent, 99%), succinonitrile (SN, Adamas, 99.0%), 2-chloroacrylonitrile (CAN, Bidepharm), tris(2-dimethylaminoethyl)amine (Me_6TREN, 99%, Amethyst), 2-chloropropionitrile (CPN, 98%, Adamas), polyacrylonitrile (PAN, 99%, Adamas, M_n = 58,200, M_w = 93,900, and $Đ$ = 1.65, **Figure S12**), anhydrous copper chloride ($CuCl_2$, 98%, Adamas), bis(trifluoromethane)sulfonimide lithium salt (LiTFSI, 99.99%, MTI) were used as received. Acrylonitrile (AN, 99.0%, Hushi) was purified by passing it through an alkaline alumina column to remove the inhibitor.

Synthesis of branched polyacrylonitrile (bPAN). (Table 1, Entry 8)

Preparation of $CuCl_2$/DMSO ([$CuCl_2$] = 10 mg/mL) stock solution: $CuCl_2$ (0.1 g, 0.744 mmol) was dissolved in DMSO (10 mL) by stirring.

Preparation of CAN/DMSO stock solution: CAN (0.064 g, 0.75 mmol, 0.0569 mL) was dissolved in DMSO (3 mL) by stirring.

Typical polymerization procedures: Polymerization was performed in a 10 mL Schlenk flask equipped with a magnetic stir bar. AN (3 mL, 2.418 g, 45.57 mmol), CPN (0.0082 g, 0.48 mmol, 0.0076 mL), the $CuCl_2$/DMSO solution (10 mg/mL, 0.0048 mmol, 0.061 mL), Me_6TREN (0.00316 g, 0.0137 mmol, 0.0035 mL), DMSO (1.5 mL) were added into the Schlenk flask. The solution was bubbled with N_2. After 10 minutes, a piece of copper wire (1 cm, Ø0.45 mm) was added and the solution was again bubbled for 5 minutes. The reaction was stirred at room temperature. Then, the CAN/DMSO solution (0.245 mol/L, 3.057 mL) was injected into the flask using a degassed

10-mL syringe with syringe pump at a rate of 0.15 mL/h (0.2 eq./h) under N_2 purging. During the polymerization process, aliquots of the solution were withdrawn at time intervals for ^{1}H nuclear magnetic resonance (NMR) and size exclusion chromatography (SEC) characterization.

Preparation of Solid-Ion Conductors (SICs)

Lithium salts-doped SN-PAN SICs were prepared by dissolving LiTFSI (0.5 M) with or without linear or branched PAN (7.5 wt%) in molten SN at 60°C for 24 h by stirring. After that the transparent mixtures were cooled to room temperature to solidify the SICs. The procedure was conducted inside an argon glovebox with a moisture content of less than 0.1 ppm.

Characterization

NMR

Quantitative ^{13}C NMR spectra were recorded on a Bruker AVANCE III HD500 spectrometer with proton decoupling without nuclear Ovenhauser enhancement, 30 s relaxation time, 256 times. Chemical shifts (δ) are reported in ppm with the solvent resonance as the internal standard (DMSO-d_6, δ: 39.5 ppm).

^{1}H NMR spectra were recorded on the same spectrometer at room temperature, and the chemical shifts are reported in ppm. ^{1}H NMR chemical shift was referenced to DMSO-d_6 (δ: 2.51 ppm).

Based on **Figure S2-S10**., the degree of branching of bPAN can be calculated using the following equations[1]:

$$\mathrm{DP_{AN}} = \frac{M_\mathrm{n}}{M_\mathrm{AN}} \tag{S1}$$

where DP_{AN} is the degree of polymerization of polyacrylonitrile, M_n is number-average molecular weight, M_{AN} is the molecular weight of acrylonitrile.

$$\mathrm{DP_{CAN}} = \frac{I_\mathrm{z}}{(I_\mathrm{y} + I_\mathrm{x})/3}\mathrm{DP_{AN}} \tag{S2}$$

where DP_{CAN} is the degree of polymerization of 2-chloroacrylonitrile; I_x, I_y, and I_z are integrated areas of H_x, H_y, and H_z, respectively, in ^{1}H NMR.

$$S_n = \frac{\mathrm{DP_{AN}}}{1 + 2\mathrm{DP_{CAN}}} \tag{S3}$$

where S_n is the spacing value.

Based on **Figure S11**, the degree of branching and the number of branches of bPAN can be calculated using the following equations:

$$\mathrm{DP_{CAN}} = \frac{I_b}{(I_a + I_\mathrm{c} + I_\mathrm{d})/3}\mathrm{DP_{AN}} \tag{S4}$$

where I_a, I_b, I_c, and I_d are integrated areas of C_a, C_b, C_c, and C_d, respectively, in ^{13}C NMR.

SEC

The number-average molecular weight (M_n), weight-average molecular weight (M_w), and dispersity ($Đ$) were determined by SEC. DMF SEC was performed using an SEC system equipped with Agilent 1260 Infinity II autosampler & quaternary pump, Shodex KD-803 + KD 805 tandem columns, and Wyatt Optilab differential refractive index (dRI) and DAWN multi-angle light scattering (MALS) detector. HPLC-grade DMF with 50 mM LiBr was used as an eluent at 1.0 mL min^{-1} at 60 °C. The dRI detector was used to measure the dn/dc ratio in-line under a 100% recovery

assumption for known injection concentrations. The system was controlled and data were processed with Wyatt Astra software.

Differential scanning calorimetry (DSC)

DSC was performed on TA Instruments DSC250 using N_2 as a purge gas at the heating rate of 10 °C min^{-1} in between −60°C and 70°C. Samples were hermetically sealed inside the Argon glovebox.

Rheological tests

Rheological behavior was investigated via oscillation experiments with a TA Instruments Discovery HR-2 rheometer at a frequency of 1 Hz over a temperature range from 0 to 80°C. Experiments were conducted with a constant strain of 40 rad/s (0.2%). Heating and cooling rates were set at 10 °C/min. Soak times for start temperatures were 60 s at 0°C (heating ramp).

Assembly of stainless steel–stainless steel (SS-SS) symmetric cells and electrochemical impedance spectroscopy (EIS) determination

LiTFSI-doped SN, SN-PAN, and SN-bPAN SICs were each pressed inside a chemical–resistant polytetrafluoroethylene (PTFE) plastic washer, with 5-mm ID, 14-mm OD, and a thickness of 200 µm. The SICs inside the PTFE washer was then sandwiched in between two SS disc shims (1-mm thick). This setup was sealed inside a CR2032 coin cell with a wave spring in place. The SS|SIC|SS cell was used to measure ionic conductivity.

EIS measurements were performed using a Solartron Analytical 1400 CellTest System from AMETEK under an open-circuit voltage condition. The alternating current voltage amplitude was

set at 10 mV, operating within a frequency range from 1 MHz to 1 Hz while maintaining temperatures at 25, 30, 35, 40, 45, and 50°C.[2]

The conductivity σ (S cm^{-1}) is calculated as:

$$\sigma = \frac{l}{R \times A} \tag{S5}$$

where l is the thickness of the electrolytes (cm); R is the resistance (Ω); and A is the area (cm^2).

The activation energy (E_A) was calculated using the Nernst-Einstein equation and the Arrhenius equation:

$$\sigma = \frac{A}{T} e^{-\frac{E_A}{kT}} \tag{S6}$$

where T is temperature (K); σ is ionic conductivity (S/cm); k is Boltzmann constant (1.380649 × 10^{-23} J/K); and A is a material-dependent preexponential constant (S·K/m).

Assembly of SS–Li asymmetric cells and linear sweep voltammetry (LSV)

In order to demonstrate the oxidation and reduction stability of the SN-bPAN electrolyte, an LSV test was conducted.[3]

LiTFSI-doped SN-bPAN SICs were each pressed inside a chemical-resistant PTFE plastic washer, with 5-mm ID, 14 mm OD, and a thickness of 200 μm. The SIC inside the PTFE washer was then sandwiched in between an SS disc shim (1-mm thick) and a Li metal disc (0.45-mm thick). This setup was sealed inside a CR2032 coin cell with a wave spring in place. LSV measurements of the SS|SIC|Li cells were conducted using a Solartron Analytical 1400 CellTest System (AMETEK).

Assembly of Li–Li symmetric cells, Li^+ transference number (t_+), and lithium stripping/plating.

LiTFSI-doped SN-bPAN SIC was pressed inside a chemical-resistant PTFE plastic washer, with 11-mm ID, 15.6 mm OD, and a thickness of 200 μm. The SIC inside the PTFE washer was then sandwiched in between two Li metal discs (0.45-mm thick). This setup was sealed inside a CR2032 coin cell with a wave spring in place.

t_+ was estimated using the AC impedance of the Li–Li symmetric cell and polarized by the DC constant potential (100 mV) polarization method. t_+ was then calculated using the Bruce-Vincent-Evans equation[4]

$$t_+ = \frac{I_s(V - I_0 \times R_0)}{I_0(V - I_s \times R_s)} \tag{S7}$$

where I_0, I_s, V, R_0, and R_s represent the initial-state current, steady-state current, applied potential, initial-state interfacial resistance, and steady-state interfacial resistance of the polymer electrolyte, respectively.

In the lithium stripping and plating process, the cell was cycled using a LAND battery test system with a current density from 0.05 to 0.2 mA cm^{-2}.

Table S1. Calculation of degree of branching.

Entry	$[AN]_0/[CAN]_0/[Initiator]_0$	M_n (kDa)	DP_{AN}	I_x	I_y	I_z	DP_{CAN}	S_n
1	90/10/1	-	-	-	-	-	-	-
2	500/8/1	153	2684	38.5	17.9	1	148	9.39
3	95/5/1	23.2	407	24.5	11.7	1	33.7	5.77
4	200/8/1	19.9	375	50.9	25.9	1	14.6	12.4
5	200/8/1	19.4	366	39.4	20.0	1	18.5	9.63
6	300/8/1	23.4	411	76.4	38.5	1	10.7	18.4
7	300/8/1	25.4	446	72.5	37.6	1	12.2	17.6
8	500/8/1	23.4	411	134	66.5	1	6.15	30.9
9	500/8/1	20.3	356	118	58.4	1	6.05	28.6
10	500/8/1	41.4	781	86.4	45.7	1	17.7	21.42

Table S2. Calculation of EA.

Entry	ln (σT)					ln A	E_A
	298 K	303 K	308 K	313 K	318 K		
SN	−0.53	−0.27	0.01	0.34	0.69	18.9	0.46
SN-PAN	−0.35	−0.06	0.20	0.46	0.70	16.3	0.4
SN-bPAN	−0.07	0.12	0.35	0.55	0.68	12.3	0.29

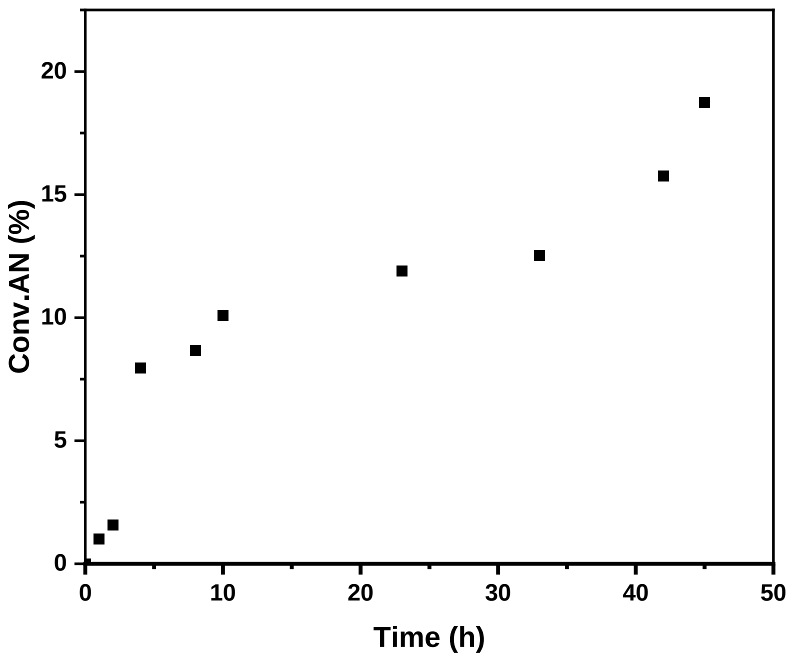

Figure S1. AN conversion versus polymerization time in the copolymerization of CAN and AN (Table 1, Entry 8).

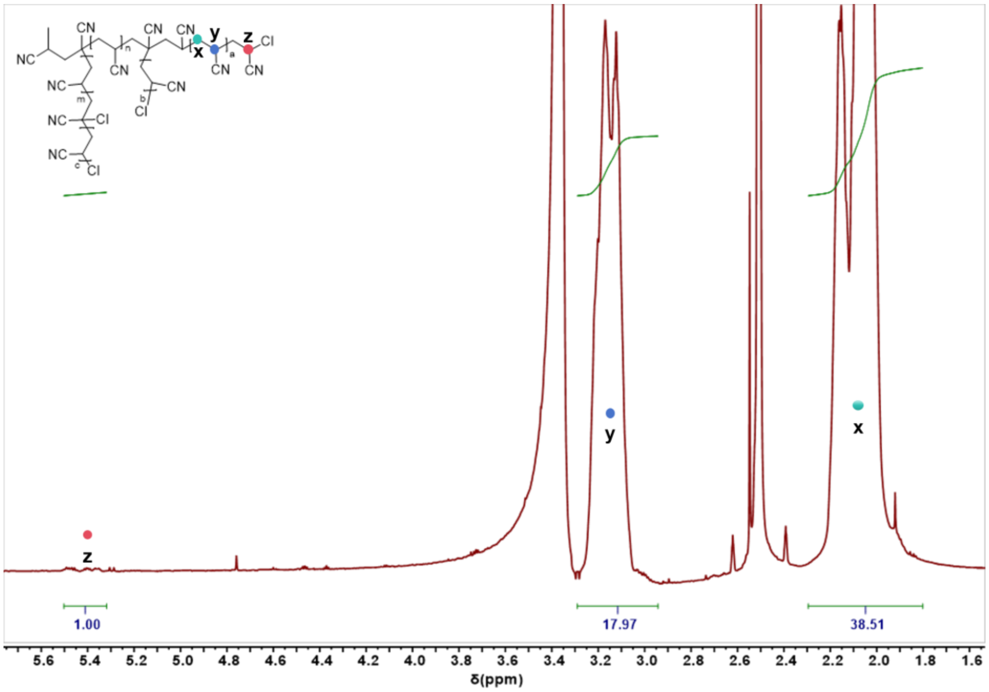

Figure S2. ^{1}H-NMR spectrum of bPAN (**Table 1**, Entry 2).

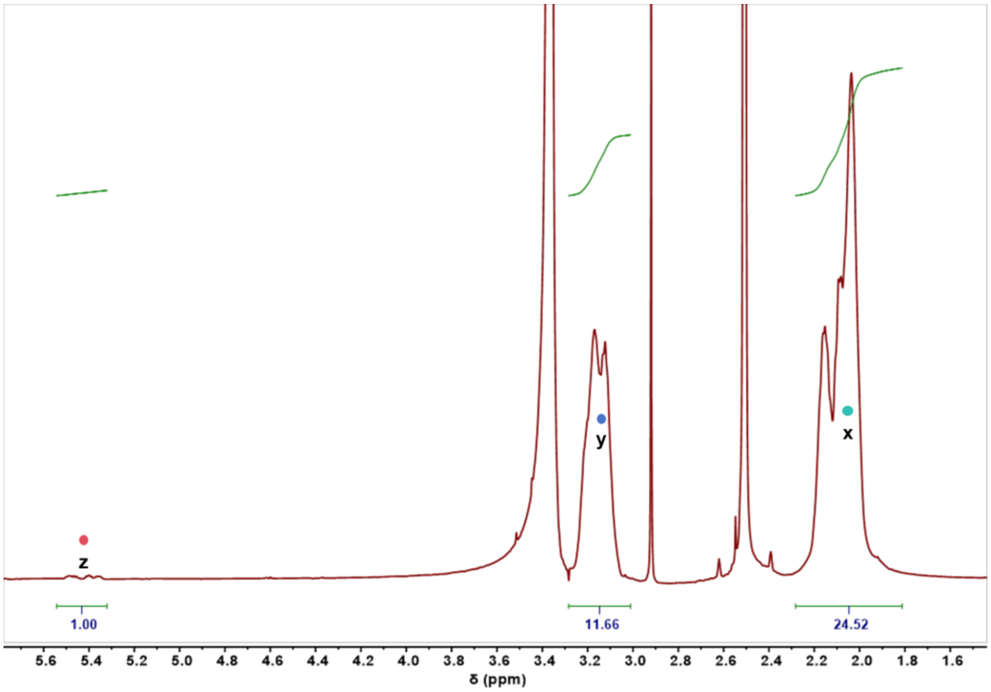

Figure S3. ^{1}H-NMR spectrum of bPAN (**Table 1**, Entry 3).

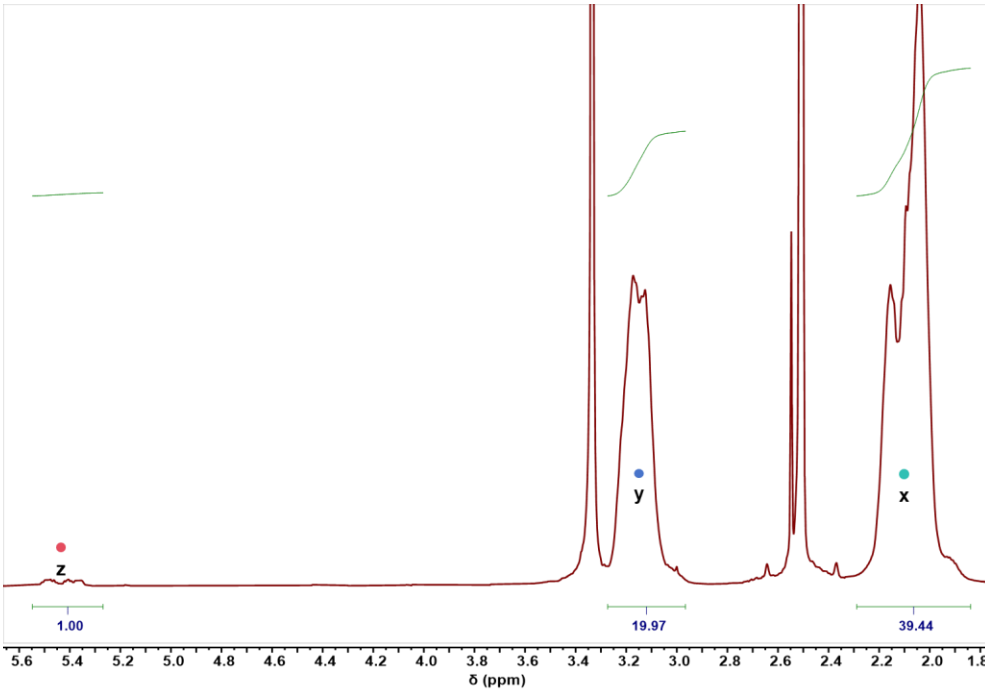

Figure S4. ^{1}H-NMR spectrum of bPAN (**Table 1,** Entry 4).

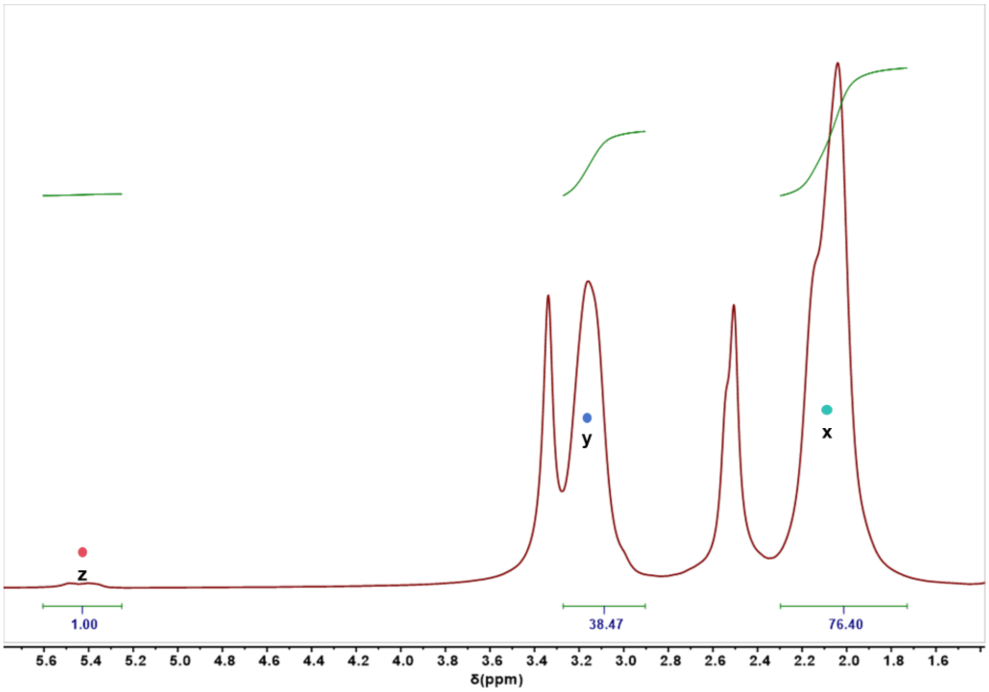

Figure S5. ^{1}H-NMR spectrum of bPAN (**Table 1**, Entry 5).

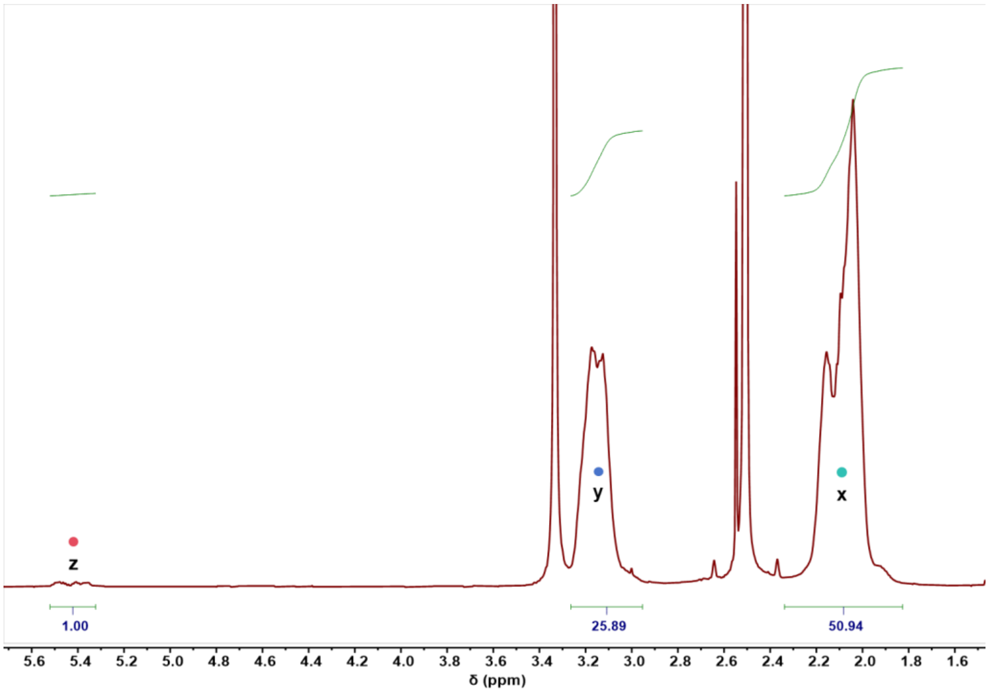

Figure S6. ^{1}H-NMR spectrum of bPAN (**Table 1**, Entry 6).

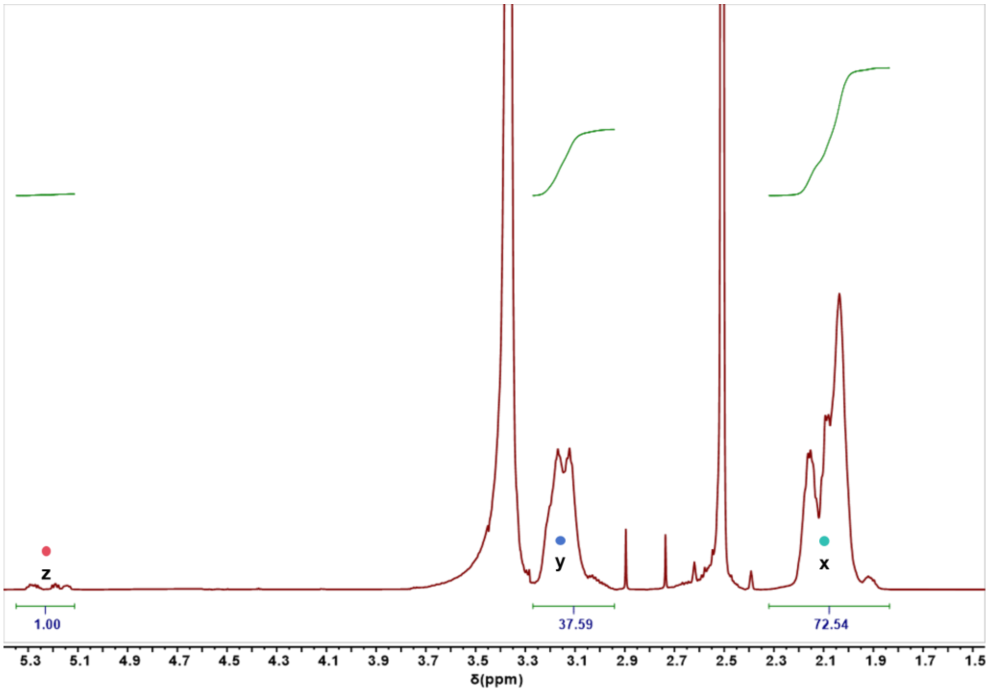

Figure S4. ^{1}H-NMR spectrum of bPAN (**Table 1**, Entry 7).

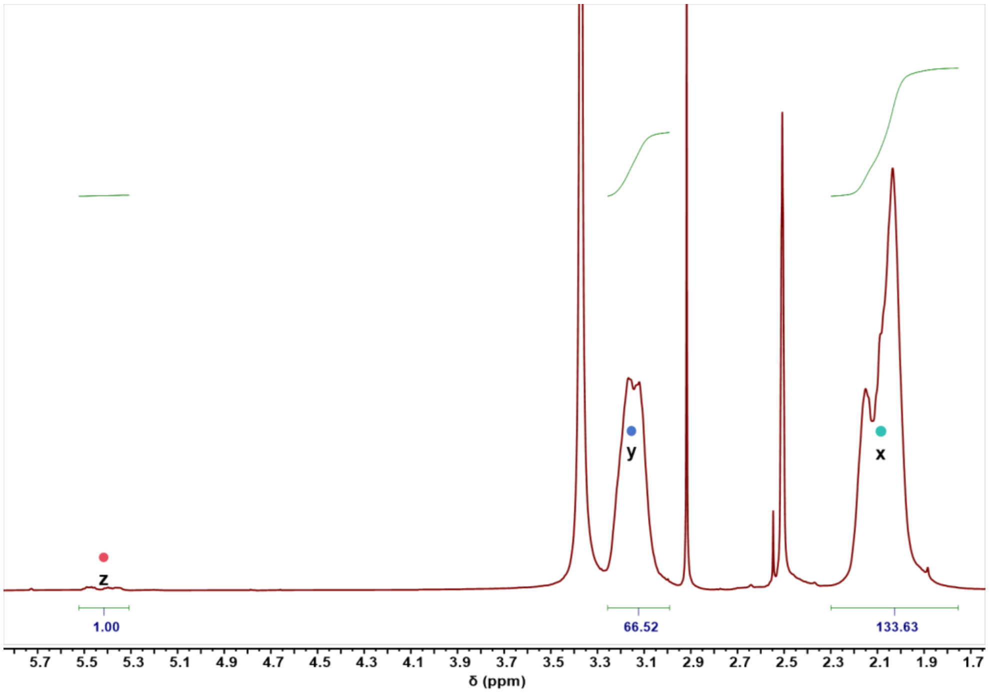

Figure S5. ^{1}H-NMR spectrum of bPAN (**Table 1**, Entry 8).

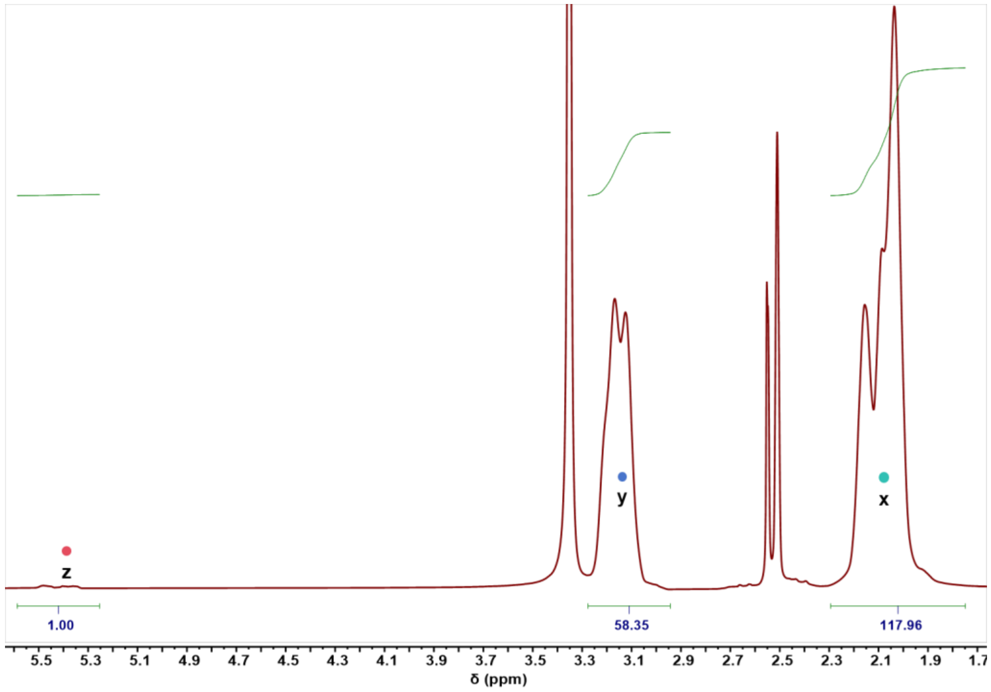

Figure S6. ^{1}H-NMR spectrum of bPAN (**Table 1**, Entry 9).

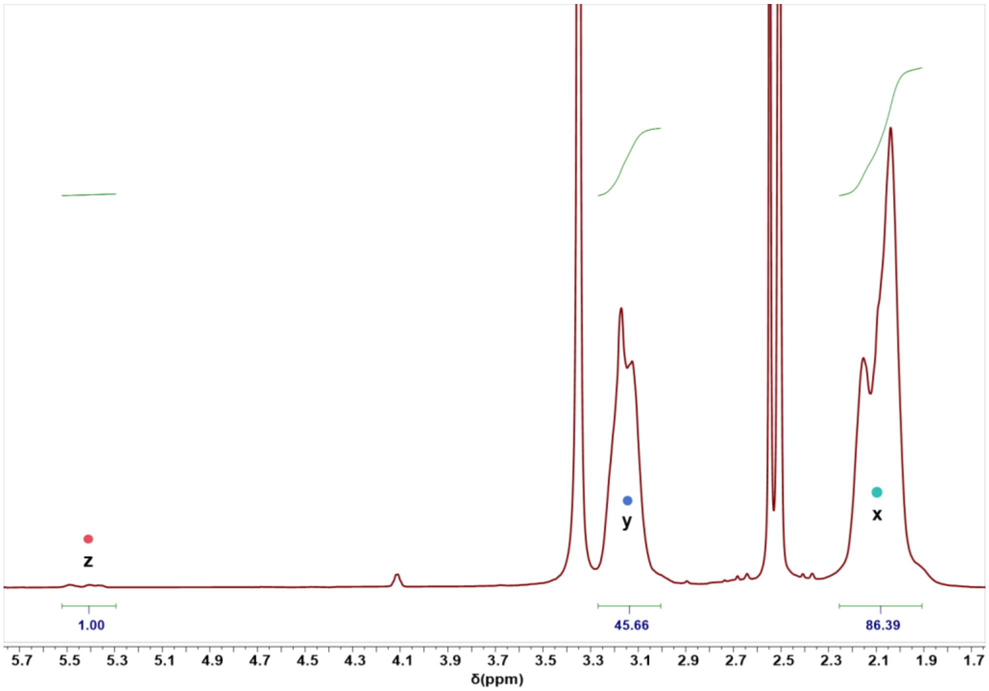

Figure S7. ^{1}H-NMR spectrum of bPAN (**Table 1**, Entry 10).

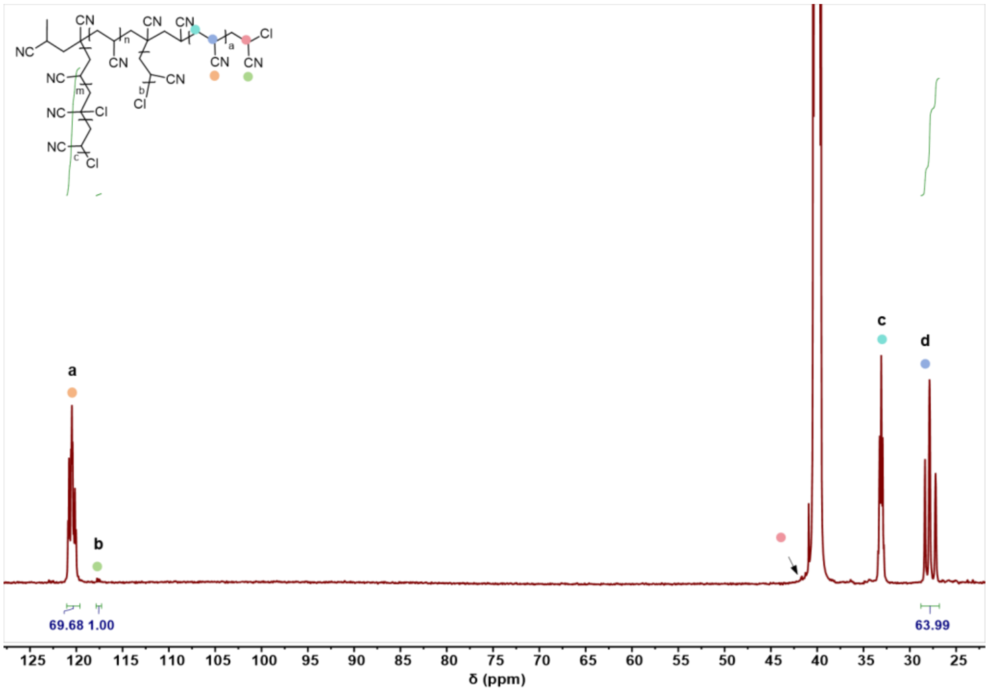

Figure S8. Quantitative ^{13}C-NMR spectrum of bPAN (**Table 1**, Entry 8).

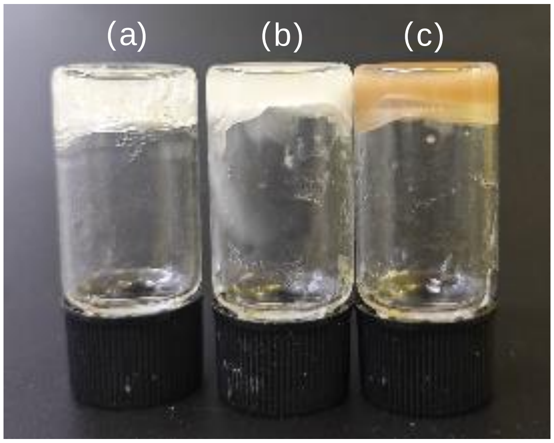

Figure S9. Digital photographs of as-prepared 0.5 M LiTFSI SICs at room temperature: (a) SN, (b) SN-PAN, and (c) SN-bPAN.

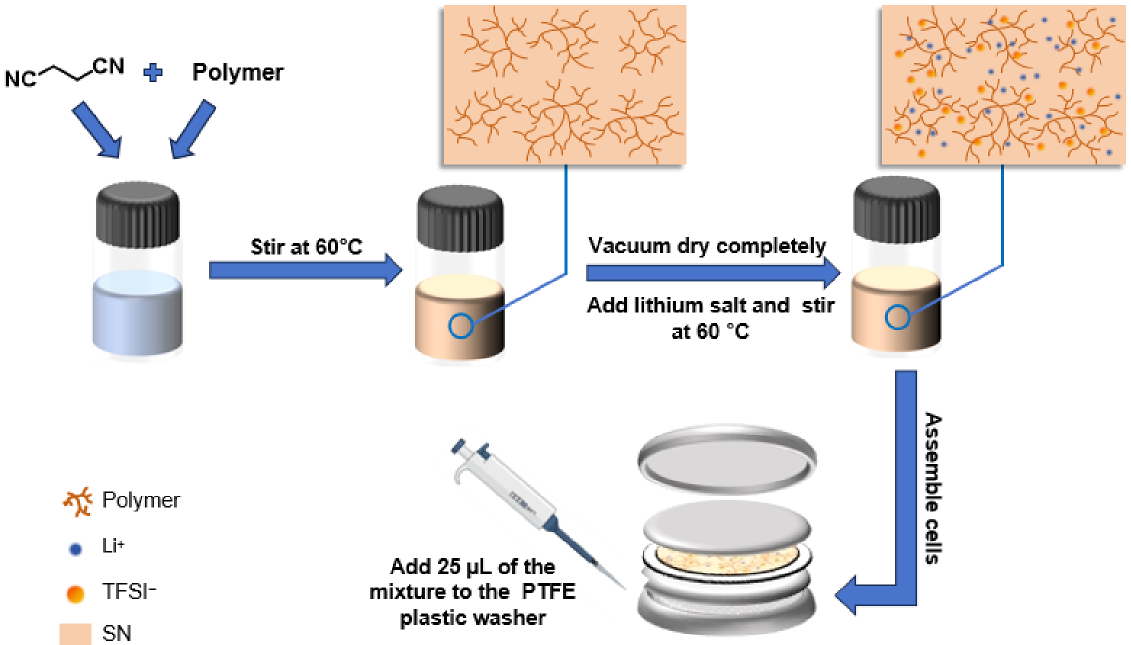

Figure S10. Preparation of electrolytes and assembly of batteries.

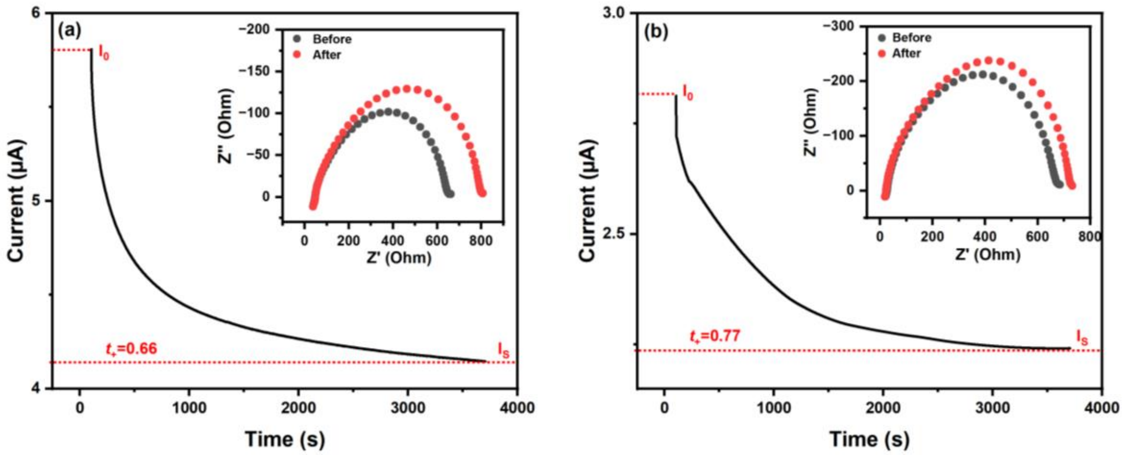

Figure S11. Li^+ transference number measurements of (a) SN, (b) SN-PAN.

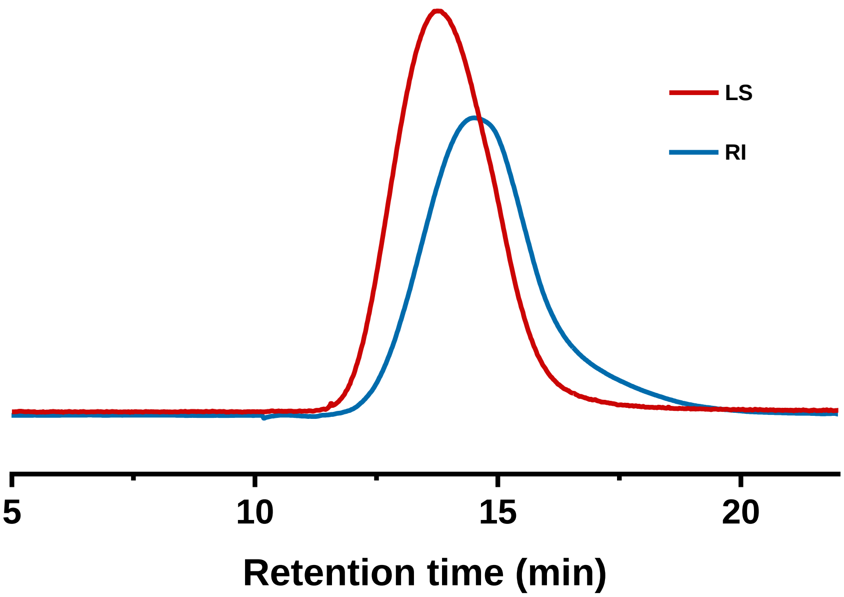

Figure S12. SEC traces of liner PAN.

Reference

(1) Cao, M.; Liu, Y.; Zhang, X.; Li, F.; Zhong, M. Expanding the toolbox of controlled/living branching radical polymerization through simulation-informed reaction design. *Chem* **2022**, *8* (5), 1460-1475. DOI: 10.1016/j.chempr.2022.02.022.

(2) Lazanas, A. C.; Prodromidis, M. I. Electrochemical Impedance Spectroscopy—A Tutorial. *ACS Meas. Sci. Au* **2023**, *3* (3), 162-193. DOI: 10.1021/acsmeasuresciau.2c00070.

(3) Zha, W.; Li, J.; Li, W.; Sun, C.; Wen, Z. Anchoring succinonitrile by solvent-Li+ associations for high-performance solid-state lithium battery. *Chem. Eng. J.* **2021**, *406*. DOI: 10.1016/j.cej.2020.126754.

(4) Chen, J.; Hu, A.; Chen, K.; Xia, Y.; Xu, W.; Li, K.; Yang, B.; Li, T.; Xu, R.; Wang, Z.; et al. Establishing ion transport channels in plastic crystal electrolytes via multifunctional cross-linked polymer matrices for stable and safe lithium metal batteries. *Nano Energy* **2025**, *139*. DOI: 10.1016/j.nanoen.2025.110959.
